# Disease risk perception, health intervention intention and their influencing factors among prehypertensive and hypertensive populations: a cross-sectional study with 1050 participants

**DOI:** 10.1186/s12889-026-28231-1

**Published:** 2026-06-25

**Authors:** Jing Zhang, Jun Liu, Juan Xu, Fengming Tang, Yang Xu, Zixun Zeng, Jing Wan

**Affiliations:** 1 Department of General Practice, Chengdu Wenjiang District People’s Hospital, Chengdu, China; 2 Department of Scientific Research and Teaching, Chengdu Wenjiang District People’s Hospital, Chengdu, China

**Keywords:** Prehypertension, Hypertension, Health Belief Model, Lifestyle, Health intervention intention, Influencing factors, Chengdu

## Abstract

**Background:**

Prehypertension and hypertension are major modifiable cardiovascular risk factors that impose a substantial global public health burden. Disease risk perception and health beliefs are critical determinants of health behaviors, yet few studies have compared the heterogeneous patterns influencing intervention intention between prehypertensive and hypertensive individuals.

**Objective:**

To compare differences in health cognition, health beliefs, lifestyle behaviors, and intervention intention between prehypertensive and hypertensive groups, and to explore heterogeneous predictors of intervention intention to inform targeted prevention strategies.

**Methodology:**

A cross-sectional study enrolled 1050 adults (525 prehypertensive, 525 hypertensive) via convenience sampling from community centers and hypertension clinics (January–December 2025). Questionnaires included demographic characteristics, lifestyle behaviors, Health Belief Model (HBM) scales, and intervention intention. Data were analyzed using SPSS 25.0 with t-tests, χ² tests, correlation analyses, and multivariate logistic regression.

**Results:**

Hypertensive patients were older, had more comorbidities, higher perceived severity, better lifestyle behaviors, and stronger intervention willingness (all *P* < 0.001). Lifestyle behaviors were positively correlated with intervention intention (*r* = 0.533, *P* < 0.01). Universal predictors included family history of hypertension, lifestyle behaviors, perceived severity, and health motivation (all *P* < 0.05). Prehypertensive individuals were primarily predicted by lifestyle behaviors (OR = 1.533, *P* < 0.001) and health motivation (OR = 1.398, *P* = 0.001); hypertensive patients by self-efficacy (OR = 1.464, *P* < 0.001), perceived susceptibility (OR = 1.163, *P* = 0.029), and lifestyle (OR = 1.089, *P* = 0.012). The prehypertension model had the highest explanatory power (Nagelkerke R²=0.849).

**Conclusions:**

Significant differences exist in health cognition, health beliefs, and intervention willingness between groups. Lifestyle behaviors serve as universal predictors of intervention intention, whereas disease perception factors play a more critical role among hypertensive patients. Targeted strategies—lifestyle optimization for prehypertension and self-efficacy enhancement for hypertension—are needed to improve self-management and reduce cardiovascular risk.

**Supplementary Information:**

The online version contains supplementary material available at https://doi.org/10.1186/s12889-026-28231-1.

## Introduction

Hypertension is a major global chronic noncommunicable disease associated with high prevalence, disability, and mortality. It affects a substantial proportion of adults worldwide and contributes to more than 10 million premature deaths each year due to cardiovascular, cerebrovascular, renal, and other related complications [1]. An estimated 1.2 billion people worldwide have uncontrolled hypertension, with particularly high burdens in middle-income countries, highlighting the urgency of targeted intervention in resource-limited settings [2]. Prehypertension, a well-established precursor to clinical hypertension, affects 25%–35% of adults globally and carries a 25%–40% risk of progression to overt hypertension within 5–10 years, representing a critical window for early cardiovascular risk prevention [3].

According to the Chinese Guidelines for the Prevention and Treatment of Hypertension (2024 revision) [4], prehypertension (also referred to as high‑normal blood pressure) is defined as systolic blood pressure (SBP) 120–139 mmHg and/or diastolic blood pressure (DBP) 80–89 mmHg, and hypertension is defined as SBP ≥ 140 mmHg and/or DBP ≥ 90 mmHg, or a confirmed diagnosis with current antihypertensive medication use.

In China, the prevalence of hypertension and prehypertension continues to rise, representing a major public health challenge. Data from a national chronic disease surveillance study (2021–2023) confirms the high prevalence of abnormal blood pressure among Chinese adults and links this burden to an increased risk of myocardial infarction, further emphasizing the need for targeted prevention and control [5]. Population-based studies from diverse settings, including rural India and urban-poor communities in Ghana, have also documented high hypertension prevalence alongside low awareness and suboptimal management, underscoring the global and heterogeneous nature of this public health challenge [6, 7]. International guidelines emphasize early intervention in prehypertension (defined as high-normal blood pressure) and structured hypertension management, with lifestyle modification as the cornerstone of preventive strategies [8, 9]. Despite the well-documented effectiveness of lifestyle modifications and pharmacological interventions in lowering blood pressure and reducing adverse cardiovascular outcomes [10–12], suboptimal adherence to health interventions and low willingness toward proactive health management remain highly prevalent in both prehypertensive and hypertensive populations [13, 14].

Previous studies have associated these behavioral gaps with insufficient disease risk perception, distorted psychological cognition, and heterogeneous health belief patterns across different blood pressure groups [15–17]. For example, low illness perception and inadequate risk awareness have been directly linked to poorer medication adherence among hypertensive patients [16], while inadequate risk perception contributes to poor health-seeking behaviors in specific at-risk populations [17]. The Health Belief Model (HBM), a classic framework in health behavior research, identifies perceived susceptibility and severity, together with perceived benefits, barriers, and self-efficacy, as core determinants of individuals’ health intervention intentions [18]. Self-efficacy, in particular, is closely associated with treatment adherence, and patient perceptions, motivations, and barriers all shape engagement in hypertension self-management [19], supporting the relevance of the HBM in understanding hypertension-related behaviors.

However, most existing studies rely on simplified assessment frameworks or focus on a single target group, lacking comprehensive comparative analyses of multi-dimensional health beliefs and lifestyle factors between prehypertensive and hypertensive individuals [20–22]. Systematic reviews indicate that many investigations only examine medication adherence among older adults or geographically restricted populations without comparing health belief profiles across blood pressure categories [23]. Others focus primarily on initial non-adherence in single-country settings without exploring multi-dimensional health belief constructs such as perceived benefits and barriers [24]. Furthermore, inadequate exploration of heterogeneous influencing mechanisms limits the generalizability and practical value of current evidence. For instance, one study investigating antihypertensive adherence only evaluated message framing and health literacy as intervention strategies, without exploring population-specific heterogeneous pathways or differences in illness perception between prehypertensive and hypertensive groups [25].

Therefore, this cross-sectional study enrolled 1050 prehypertensive and hypertensive participants, using a comprehensive assessment tool covering demographic characteristics, clinical indicators, the Health Belief Model scale, and lifestyle factors, with multivariable statistical approaches for in-depth comparative analysis. The objectives were to: (1) clarify differences in demographics, health cognition, health beliefs, lifestyle, and intervention intention between the two groups; (2) explore correlations between key indicators and health intervention intention; (3) identify independent influencing factors and heterogeneous patterns of intervention intention in different subgroups; (4) provide evidence to support the development of targeted, population-specific strategies for hypertension prevention and management in community public health practice.

## Methods

### Study design

This population‑based cross‑sectional study was conducted in Chengdu City, Sichuan Province, Southwest China, between January and December 2025. Data were collected once from each participant during a single study visit. The study protocol was approved by the Medical Ethics Committee of Chengdu Wenjiang District People‘s Hospital (Approval ER‑2024‑007). Results were reported based on standardized questionnaires and physical measurements (blood pressure, height, weight).

### Study population and grouping criteria

Chengdu is a major urban center with a permanent resident population of 21.47 million as of 2025, representing a typical large metropolitan area in western China [26]. The source population comprised community-dwelling adults aged 18 years and older living in Chengdu. Participants were recruited through convenience sampling from the Department of General Practice and the Department of Cardiovascular Medicine of Wenjiang District People’s Hospital, as well as from five community health service centers within the Wenjiang District Medical Alliance in Chengdu, between January and December 2025. Trained research assistants approached potential participants during routine health visits or community health promotion activities.Participants were stratified into two groups according to the Chinese Guidelines for the Management of Arterial Hypertension (2024) [4]:

- Prehypertension group: systolic blood pressure (SBP) 120–139 mmHg and/or diastolic blood pressure (DBP) 80–89 mmHg, with no prior diagnosis of hypertension or use of antihypertensive medications.
- Hypertension group: SBP ≥ 140 mmHg and/or DBP ≥ 90 mmHg, or a confirmed diagnosis of hypertension with current antihypertensive medication use.

### Inclusion and exclusion criteria

#### Inclusion criteria

(1) age ≥ 18 years; (2) clear consciousness and normal communication ability; (3) voluntary participation and willingness to provide written informed consent; (4) ability to complete the questionnaire independently or with assistance.

#### Exclusion criteria

(1) secondary hypertension (e.g., due to renal artery stenosis, primary aldosteronism, or other endocrine disorders); (2) severe dysfunction of the heart (New York Heart Association class III or IV), liver (Child-Pugh class C), kidney (estimated glomerular filtration rate < 30 mL/min/1.73 m²), or brain (history of major stroke with residual neurological deficits); (3) mental disorders or cognitive impairment that would preclude informed consent or questionnaire completion; (4) pregnancy or lactation; (5) current participation in another interventional study.

### Sample size determination

The sample size was determined based on a two-group comparison of proportions. With a significance level of 0.05 (two-sided), a power of 0.80, and assuming a 10%-point difference in the primary outcome (willingness to undergo health interventions) between the prehypertension and hypertension groups, the required sample size was approximately 365 participants per group. After adding 20% to account for potential missing data or incomplete responses, the target sample size was 438 participants per group. To enhance statistical power for secondary analyses and ensure robustness of subgroup comparisons, we ultimately recruited 525 participants per group, yielding a total of 1,050 participants.

The equal sample sizes per group were a design target to ensure balanced statistical power for subgroup comparisons; no individual matching was performed. Demographic differences between groups were adjusted for in multivariate analyses.

According to the China Hypertension Survey, the prevalence of hypertension among Chinese adults is approximately 27.5% [6], and more recent data from 2021 to 2022 show a prevalence of 31.6% [27]. The prevalence of prehypertension (or high-normal blood pressure) has been reported as 43.8% among young and middle-aged adults [27]. Furthermore, the Report on Population Health Status and Key Diseases in Sichuan Province (2024 Health White Paper), issued by the Sichuan Provincial Health Commission, indicates that data from the 2023 chronic disease risk factor surveillance revealed a hypertension prevalence of 33.55% among residents aged ≥ 18 years in Sichuan Province [28], which is higher than the national prevalence.These epidemiological data indicate that both conditions affect substantial proportions of the Chinese population, supporting the generalizability of our study sample.

### The survey instrument

The following instruments were used to collect data from all participants.

#### General information questionnaire

A self-designed questionnaire was used to collect demographic characteristics, clinical indicators, and health cognition. The clinical indicators included family history of hypertension, comorbidities, and self‑reported health status. Health cognition was assessed by recognition of hypertension as a lifelong disease and awareness of preventive methods.

#### Lifestyle scale

This scale was adapted from the “Health Examination Self-Assessment Questionnaire” (Expert Consensus on Basic Items of Health Examination) and further modified by Tan et al. (in Chinese) [29]. It comprises 30 items across six dimensions: dietary habits, food intake, physical activity, smoking, alcohol consumption, and mental stress. The scale showed good internal consistency (Cronbach‘s α = 0.89). Items are scored on a − 5 to + 5 scale with positive or negative coding (e.g., smoking and alcohol are reverse-coded). Higher scores indicate healthier behaviors. Dimension scores are summed and transformed to a total score ranging from 0 to 100, categorized as unsatisfactory (< 70), average (70–<90), or ideal (≥ 90). The scale was pilot-tested in 50 Chinese adults before formal use.

#### Health belief model (HBM) scale

This modified and validated scale (Cronbach’s α = 0.85) was based on the Champion‘s Health Belief Model Scale and comprised six dimensions: perceived susceptibility, perceived severity, perceived benefits, perceived barriers, health motivation, and self-efficacy [30]. The HBM items assess beliefs about hypertension (e.g., “I am at risk of developing hypertension”). Each dimension is scored separately; higher scores reflect stronger endorsement of the respective belief (e.g., higher perceived severity means greater belief that hypertension is serious). For the perceived barriers dimension, reverse scoring was applied. The scale was independently translated and back-translated by two bilingual researchers, followed by a pilot test in 30 participants to ensure clarity and cultural appropriateness. The HBM scale has been previously validated in Chinese chronic disease populations, including patients with hypertension [30–32].

Health Intervention Intention Scale: This scale assessed participants‘ willingness to undergo both core interventions (general health management) and specific interventions (medication, diet, exercise, and stress/sleep adjustment). Responses were originally categorical (“very willing” / “willing” / “unwilling”), which were collapsed into a binary variable (willing vs. unwilling) for logistic regression analysis. Unlike health motivation (general health concern) or self-efficacy (confidence to act), intervention intention directly captures the willingness to perform specific actions (e.g., “I am willing to take antihypertensive medication as prescribed”).

### Data collection

Trained investigators conducted face-to-face standardized interviews with all participants. Blood pressure was measured three times at 5-minute intervals using an upper-arm electronic sphygmomanometer, and the average of the three readings was recorded for analysis. Height and weight were measured following standard protocols to calculate body mass index (BMI). To minimize data entry errors, all questionnaire data were double-entered independently by two data entry staff using EpiData software version 3.1, followed by consistency checks.

### Statistical analysis

Statistical analyses were performed using IBM SPSS Statistics version 25.0. Continuous variables with a normal distribution were expressed as mean ± standard deviation (SD) and compared between the two groups using the independent-samples t-test. Categorical variables were presented as frequencies (percentages) and compared using Pearson’s chi-square (χ²) test.

#### Variable selection for regression analysis

Variables with *P* < 0.05 in univariate analysis or those reported as important predictors in the literature (e.g., age, sex, family history of hypertension, HBM dimensions, lifestyle score) were entered into the multivariate logistic regression model. The coding scheme for all categorical variables used in the logistic regression analysis is provided in Supplementary Table 2.

#### Multicollinearity assessment

We assessed variance inflation factors (VIF) for all predictors; all VIF values were < 2.5, indicating no significant multicollinearity.

#### Outlier check

We examined studentized residuals and Cook’s distance; no influential outliers were detected.

Spearman‘s rank correlation coefficient was used to examine associations between core indicators and intervention intention because the Shapiro‑Wilk test indicated that several variables (e.g., lifestyle score, HBM dimensions) were not normally distributed (*P* < 0.05). Spearman’s correlation is a non‑parametric method that does not assume normality.

A binary multivariate logistic regression analysis was conducted with intervention intention as the dependent variable (coded as 0 = “unwilling”, 1 = “willing/very willing”). Independent variables were entered in three sequential blocks:

- Block 1: demographic characteristics;
- Block 2: dimensions of the Health Belief Model (HBM);
- Block 3: lifestyle score.

Model fit was evaluated using the Omnibus test, the Hosmer–Lemeshow goodness-of-fit test, and Nagelkerke’s R². All statistical tests were two-sided, and a P-value < 0.05 was considered statistically significant.

#### Missing data

Participants with missing data on the primary outcome or key predictors (less than 3% of the sample) were excluded from the respective analyses. No multiple imputation was performed due to the low proportion of missing data.

## Results

**Fig. 1:**
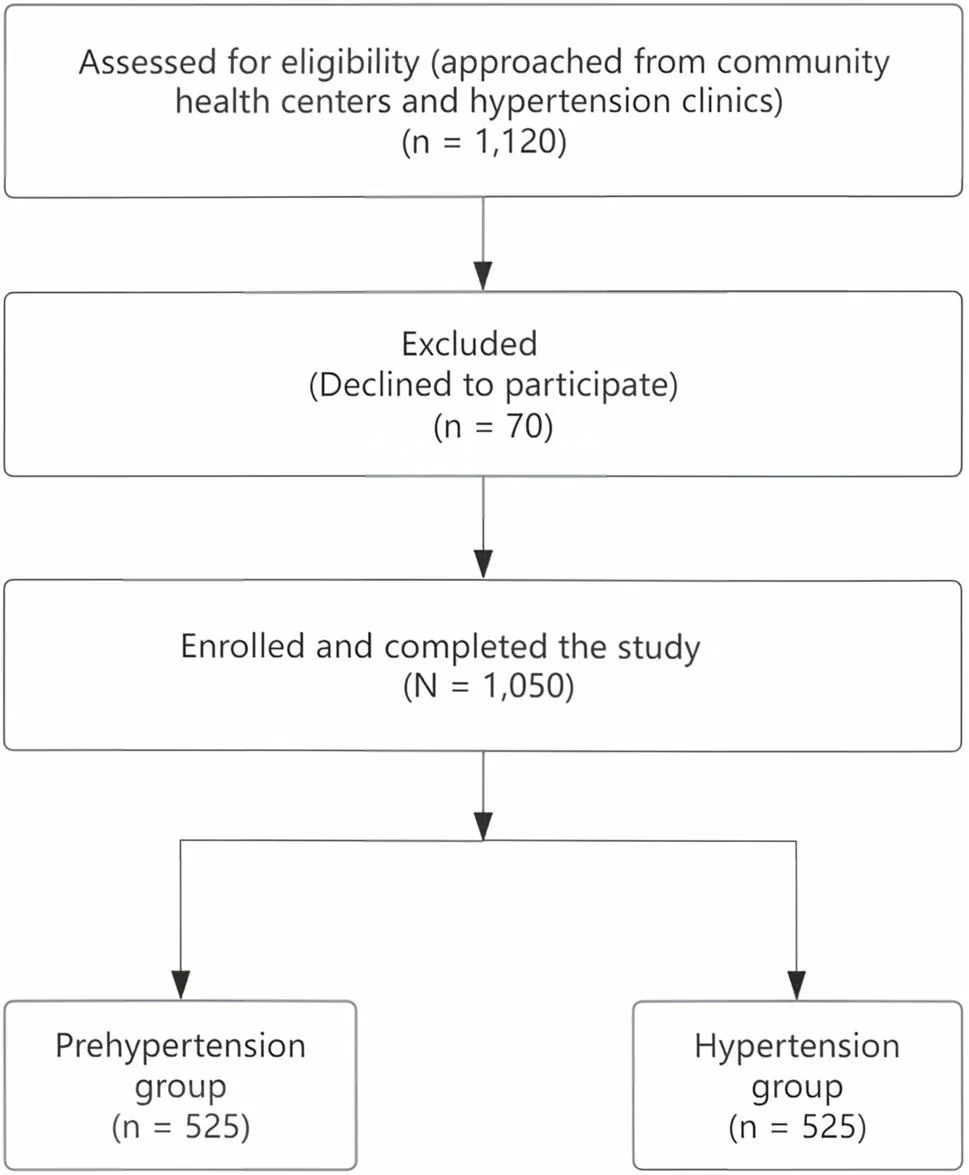
Participant flow diagram. Of 1,120 individuals approached, 70 declined to participate. The remaining 1,050 completed the study and were divided into prehypertension (*n* = 525) and hypertension (*n* = 525) groups

Analytical sample and participant flow. A total of 1,050 participants were included in the final analysis (525 prehypertensive, 525 hypertensive). No participants were excluded due to missing data, as all questionnaires were completed in full. Figure 1 presents the participant flow diagram: 1,120 individuals were approached, 70 declined to participate, and 1,050 completed the study. Missing data were minimal: less than 1% of participants had missing values for any single variable (e.g., lifestyle score: *n* = 3, 0.3%; HBM dimensions: *n* = 2–5 per dimension, 0.2–0.5%; intervention intention: *n* = 0).

### Demographic and clinical characteristics

Except for BMI (*P* = 0.444), the two groups showed significant differences in all baseline indicators (all *P* < 0.001, Table 1). The hypertensive group had higher age (62.28 ± 13.69 vs.46.77 ± 14.26 years), male proportion (72.4% vs.60.2%), comorbid chronic diseases (44.0% vs.7.6%), family history of hypertension (62.5% vs.42.7%) and recognition of hypertension as a lifelong disease (91.8% vs.58.1%). The prehypertensive group had a higher proportion of college or above education (45.1% vs.28.6%), government/institution occupation (45.3% vs.13.9%) and family monthly income ≥ 5000 CNY (80.4% vs.52.9%). The hypertensive group also had higher lifestyle scores (75.75 ± 7.74 vs.68.31 ± 11.82) and core intervention willingness (90.9% vs.68.0%). Table 1 now includes a column for the total population (*N* = 1,050).

**Table 1:** Demographic and baseline characteristics of study participants

Characteristics	Total (*n* = 1050)	Prehypertension (*n* = 525)	Hypertension (*n* = 525)	Test (t/χ²)	*P*-value
Age (years)	54.53 ± 14.56	46.77 ± 14.26	62.28 ± 13.69	t = 17.97	< 0.001
Gender				χ² = 17.46	< 0.001
Male	696 (66.3)	316 (60.2)	380 (72.4)		
Female	354 (33.7)	209 (39.8)	145 (27.6)		
Education level				χ² = 33.17	< 0.001
Junior high or below	305 (29.0)	142 (27.0)	163 (31.0)		
High school/technical	358 (34.1)	146 (27.8)	212 (40.4)		
College or above	387 (36.9)	237 (45.1)	150 (28.6)		
Occupation				χ² = 125.47	< 0.001
Government/institution	311 (29.6)	238 (45.3)	73 (13.9)		
Enterprise/business	386 (36.8)	157 (29.9)	229 (43.6)		
Retired	353 (33.6)	130 (24.8)	223 (42.5)		
Family monthly income (CNY)				χ² = 93.75	< 0.001
< 2500	48 (4.6)	20 (3.8)	28 (5.3)		
2500–4999	302 (28.8)	83 (15.8)	219 (41.7)		
5000–7500	451 (43.0)	264 (50.3)	187 (35.6)		
≥ 7500	249 (23.7)	158 (30.1)	91 (17.3)		
Marital status				χ² = 31.26	< 0.001
Unmarried	124 (11.8)	68 (13.0)	56 (10.7)		
Married/ cohabiting	772 (73.5)	412 (78.5)	360 (68.6)		
Widowed/divorced	154 (14.7)	45 (8.6)	109 (20.8)		
Living status				χ² = 36.87	< 0.001
Living alone	201 (19.1)	82 (15.6)	119 (22.7)		
With family (non-parents/children)	404 (38.5)	172 (32.8)	232 (44.2)		
With children/parents	445 (42.4)	271 (51.6)	174 (33.1)		
BMI (kg/m²)	25.03 ± 4.02	24.93 ± 3.51	25.12 ± 4.49	t = -0.77	0.444
Comorbid chronic diseases				χ² = 181.69	< 0.001
None	779 (74.2)	485 (92.4)	294 (56.0)		
Diabetes	227 (21.6)	32 (6.1)	195 (37.1)		
Other diseases	44 (4.2)	8 (1.5)	36 (6.9)		
Family history of hypertension (Yes)	551 (52.5)	224 (42.7)	327 (62.5)	χ² = 41.43	< 0.001
Lifestyle score	72.03 ± 10.71	68.31 ± 11.82	75.75 ± 7.74	t = -12.05	< 0.001
Core intervention willingness (Willing)	834 (79.4)	357 (68.0)	477 (90.9)	χ² = 83.93	< 0.001

Comparison of demographic characteristics, clinical indicators, lifestyle scores, and core intervention willingness between prehypertensive (*n* = 525) and hypertensive (*n* = 525) groups. Data are presented as mean ± SD for continuous variables and n (%) for categorical variables. *P*-values were calculated using independent-samples t-test for continuous variables and Pearson’s χ² test for categorical variables. Statistically significant differences (*P* < 0.05) were observed for all indicators except BMI

*BMI *Body Mass Index

### Hypertension awareness and intervention willingness

**Fig. 2:**
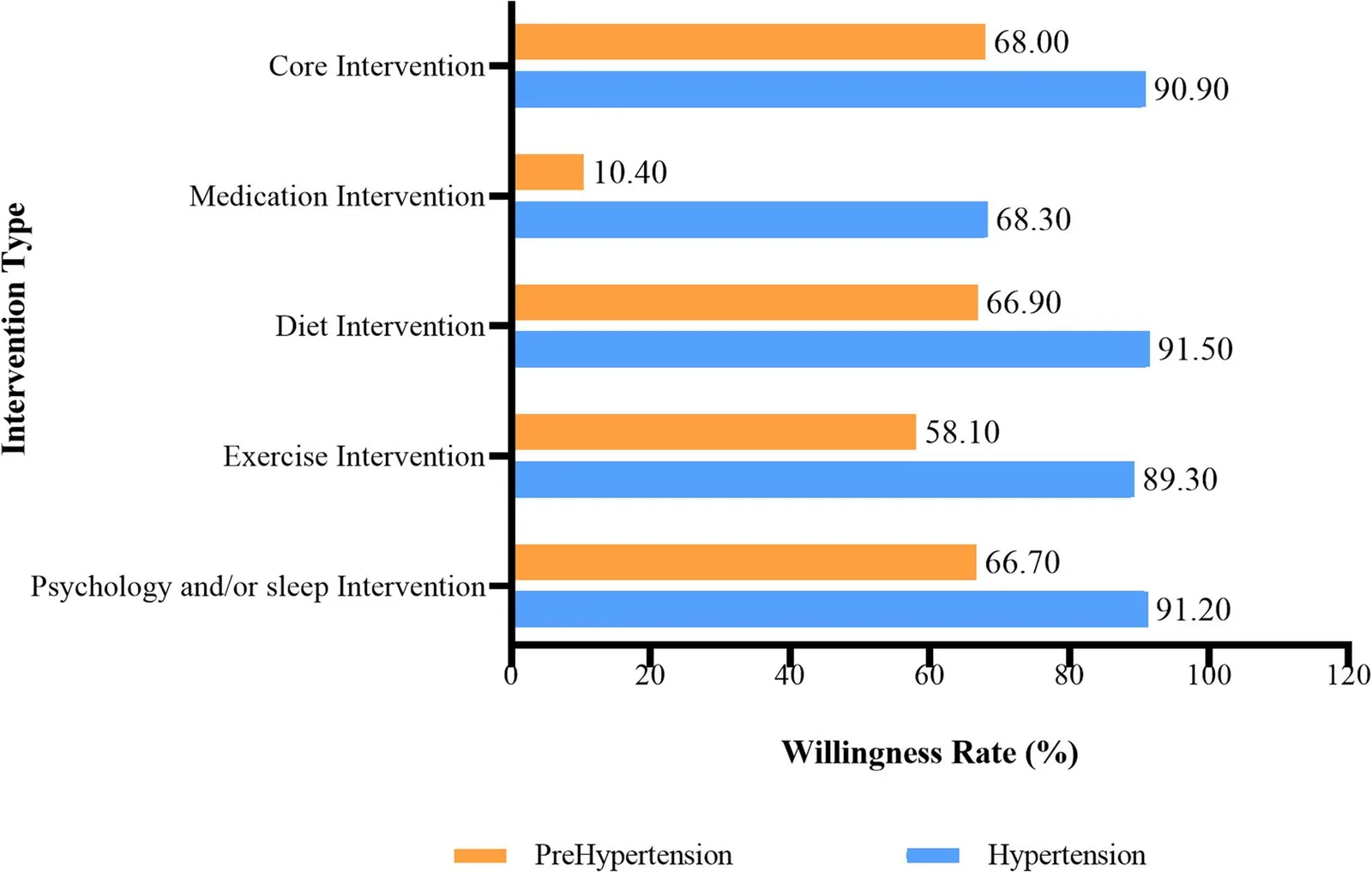
Comparison of health intervention willingness across five intervention types. Comparison of health intervention willingness rates (%) between prehypertensive (*n*=525) and hypertensive (*n*=525) groups across five intervention types: core intervention, medication intervention, diet adjustment, exercise intervention, and stress/sleep adjustment. Hypertensive patients consistently showed higher willingness than prehypertensive individuals across all dimensions, with the largest gap observed for medication intervention (68.3% vs. 10.4%). Data are presented as percentages of participants who reported being "willing" or "very willing"

All indicators of hypertension awareness and intervention willingness differed significantly between the two groups (all *P* < 0.001). Correct recognition of the 140/90 mmHg diagnostic threshold was similar (55.4%vs.54.5%), but the hypertensive group had a higher recognition rate of 130/80 mmHg (20.2% vs.2.3%). The hypertensive group also had 100.0% awareness of all hypertension risk factors and prevention methods, compared with 91.0% and 96.2% in the prehypertensive group. The hypertensive group showed significantly higher willingness in all intervention dimensions (as shown in Fig. 2): core intervention (90.9% vs.68.0%), medication intervention (68.3% vs.10.4%), diet adjustment (91.5% vs.66.9%), exercise intervention (89.3% vs.58.1%), and stress/sleep adjustment (91.2% vs.66.7%).

Figure 2 presents the comparison of intervention willingness across five intervention types. Hypertensive patients consistently showed higher willingness than prehypertensive individuals across all dimensions, with the largest gap observed for medication intervention (68.3% vs. 10.4%).

### HBM dimension scores

**Fig. 3:**
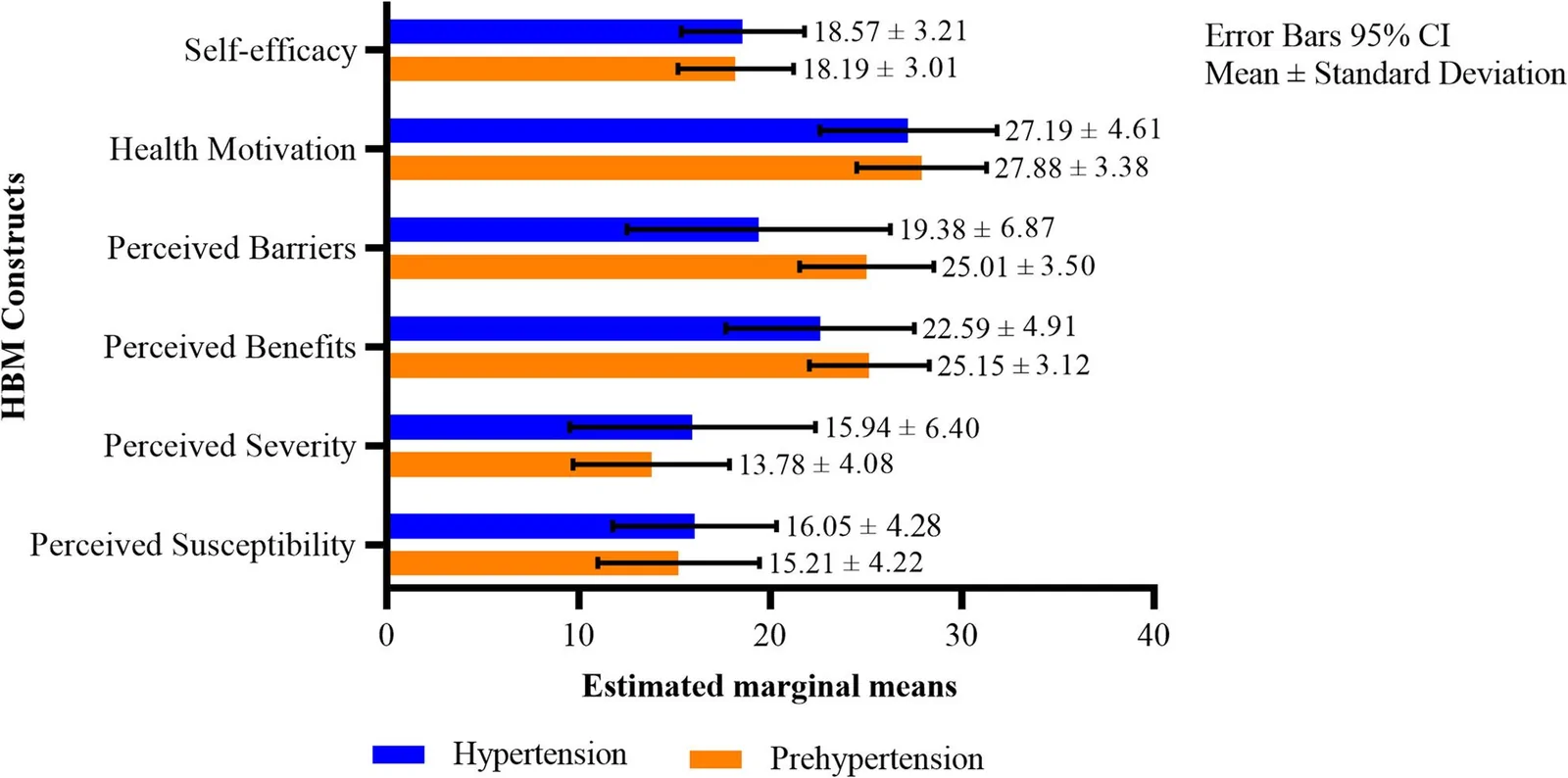
Comparison of HBM dimension scores between prehypertensive and hypertension groups. Bar chart comparing mean scores of six HBM dimensions (perceived susceptibility, perceived severity, perceived benefits, perceived barriers, health motivation, and self-efficacy) between prehypertensive (*n *= 525) and hypertensive (*n *= 525) groups. Error bars represent standard deviations (SD)

Figure 3 illustrates the differences in HBM dimension scores between the two groups. Hypertensive patients scored higher than prehypertensive individuals in perceived susceptibility (16.05 ± 4.28 vs. 15.21 ± 4.22) and perceived severity (15.94 ± 6.40 vs. 13.78 ± 4.08). However, prehypertensive individuals scored higher than hypertensive patients in perceived benefits (25.15 ± 3.12 vs. 22.59 ± 4.91), perceived barriers (25.01 ± 3.50 vs. 19.38 ± 6.87), and health motivation (27.88 ± 3.38 vs. 27.19 ± 4.61). Self-efficacy scores were comparable between the two groups (18.57 ± 3.21 vs. 18.19 ± 3.22).Although some error bars overlap, independent-samples *t*-tests confirmed significant differences between the two groups for all six dimensions (all *P* < 0.05; see Supplementary Table 4 for detailed statistics).

### Correlation analysis results

Lifestyle score was the strongest positive correlate of health intervention willingness in all populations (all *P* < 0.01), with the highest correlation in the prehypertensive group (*r* = 0.730, Table 2). Health motivation were positively correlated with intervention willingness in both groups (all *P* < 0.01), with significantly higher coefficients in the hypertensive group (health motivation *r* = 0.213 vs.0.147); Self-efficacy showed a weak positive correlation in the total population (*r* = 0.152, *P* < 0.01), and a moderate positive correlation in the hypertension group (*r* = 0.288, *P* < 0.01). No significant correlation in the prehypertension group (*P* > 0.05). Perceived barriers were negatively correlated with intervention willingness in the total population (*r*=-0.100, *P* < 0.01), while perceived susceptibility was only negatively correlated in the total population (*r*=-0.062, *P* < 0.05) with no significant correlation in either subgroup (*P* > 0.05). Perceived severity and benefits showed no significant correlations with intervention willingness in any group (all *P* > 0.05).

**Table 2:** Correlation between core indicators and health intervention willingness

Correlated Variables	Total Population *r* (*P*-value)	Prehypertension *r* (*P*-value)	Hypertension *r* (*P*-value)
Lifestyle Questionnaire Score	0.533 ^**^	0.730 ^**^	0.133 ^**^
Perceived Susceptibility	-0.062^*^	0.000	0.070
Perceived Severity	-0.058	-0.037	0.053
Perceived Benefits	-0.018	-0.077	0.099 ^*^
Perceived Barriers	-0.100 ^**^	0.074	-0.014
Health Motivation	0.143 ^**^	0.147 ^**^	0.213 ^**^
Self-efficacy	0.152 ^**^	0.047	0.288 ^**^

Spearman's rank correlation coefficients (r) between lifestyle questionnaire score, HBM dimensions (perceived susceptibility, perceived severity, perceived benefits, perceived barriers, health motivation, self-efficacy), and health intervention willingness in the total population (*N*=1,050), prehypertension group (*n*=525), and hypertension group (*n*=525)

^*^P < 0.05; ^**^P < 0.01

**Fig. 4:**
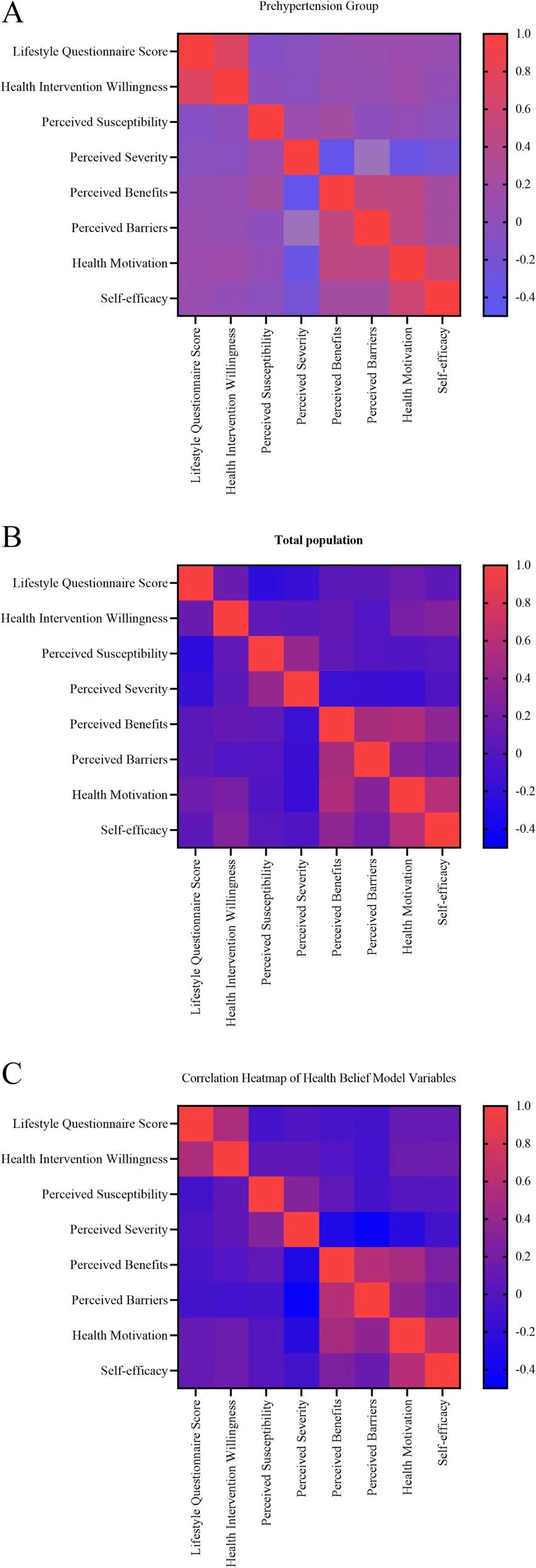
Correlation heatmaps of HBM dimensions and health intervention intention. **A **Prehypertension group (*n*=525); **B** Hypertension group (*n*=525); **C** Total population (*N*=1,050). The color gradient represents the strength of Spearman's rank correlation coefficient (r), with darker colors indicating stronger correlations. *P < 0.05; **P < 0.01

The correlation patterns between HBM variables and health intervention intention are visualized in Fig. 4. In the prehypertension group (Fig. 4A), lifestyle score showed a strong positive correlation with intervention willingness (*r* = 0.730). In the hypertension group (Fig. 4B), self-efficacy demonstrated a moderate positive correlation (*r* = 0.288). The total population analysis (Fig. 4C) confirmed lifestyle score as the strongest correlate (*r* = 0.533).

### Multivariate logistic regression analysis

#### Model fit

**Fig. 5:**
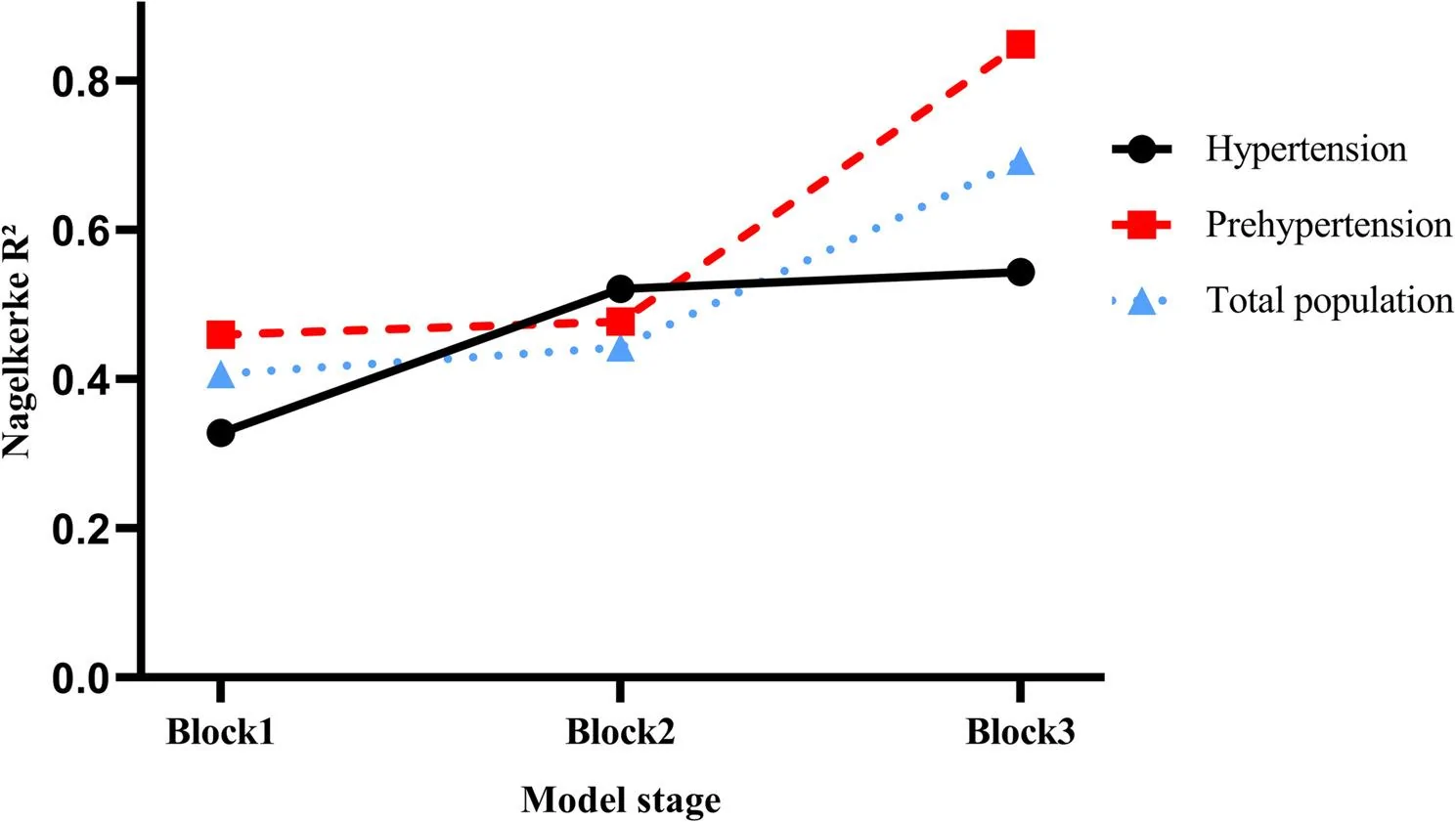
Nagelkerke R² values of stepwise logistic regression models. Nagelkerke R² values for total population (*N *= 1,050), prehypertension group (*n *= 525), and hypertension group (*n *= 525) across three sequential blocks. Block 1: demographic and clinical variables; Block 2: addition of HBM dimensions; Block 3: addition of lifestyle score

All regression models had acceptable fit (Hosmer-Lemeshow *P* > 0.05) and stable convergence. The incremental explanatory power of each variable block is shown in Fig. 5. For the prehypertensive group, the model demonstrated the highest explanatory power, with Nagelkerke R² increasing from 0.460 in Block 1 (demographic and clinical variables) to 0.477 in Block 2 (adding HBM dimensions), and substantially to 0.849 in Block 3 (adding lifestyle score). Lifestyle score contributed the largest increment (ΔR² = 0.372, *P* < 0.001), while HBM dimensions showed no incremental effect (ΔR² = 0.017, *P* = 0.165), indicating that lifestyle factors were strongly associated with intervention intention in this subgroup. For the hypertensive group, the model showed moderate explanatory power, with Nagelkerke R² increasing from 0.328 in Block 1 to 0.521 in Block 2, and modestly to 0.544 in Block 3. HBM dimensions were the main contributors (ΔR² = 0.193, *P* < 0.001), while lifestyle score added only a small incremental effect (ΔR² = 0.023, *P* = 0.010), suggesting that health beliefs were important correlates of intervention intention among hypertensive patients.For the total population, the model exhibited good explanatory power, with Nagelkerke R² increasing from 0.407 in Block 1 to 0.443 in Block 2, and further to 0.693 in Block 3. Both HBM dimensions (ΔR² = 0.036, *P* < 0.001) and lifestyle score (ΔR² = 0.250, *P* < 0.001) contributed significantly to model fit, indicating that a combination of health beliefs and lifestyle factors underpins intervention intention in the general population.

### Predictors in the total population

As shown in Table 3, with all categorical variables using the last category as the reference group, demographic factors had no significant effects on health intervention intention in the total population (all *P* > 0.05). Independent positive factors included family history of hypertension (OR = 3.043, 95% CI = 1.725–5.369, *P* < 0.001), lifestyle score (OR = 1.269, 95% CI = 1.218–1.323, *P* < 0.001), perceived severity (OR = 1.078, 95% CI = 1.014–1.146, *P* = 0.017), and health motivation (OR = 1.128, 95% CI = 1.029–1.236, *P* = 0.010). Perceived susceptibility showed a borderline association (OR = 1.074, 95% CI = 0.998–1.155, *P* = 0.056). Perceived benefits, perceived barriers, and self-efficacy were not significantly associated with intervention intention (all *P* > 0.05). Complete regression results, including all non-significant demographic variables, are provided in Supplementary Table 3. Stratified analysis revealed heterogeneous patterns between the two groups (Supplementary Table 1). For the prehypertensive group, independent positive factors for health intervention intention included family history of hypertension (OR = 22.557, 95% CI: 7.007–72.622, *P* < 0.001), lifestyle score (OR = 1.533, 95% CI: 1.388–1.692, *P* < 0.001), health motivation (OR = 1.398, 95% CI: 1.150–1.700, *P* = 0.001), age group 40–59 years (OR = 6.710, 95% CI: 1.154–39.014, *P* = 0.034), and occupation in government/institution (OR = 3.932, 95% CI: 1.076–14.364, *P* = 0.038). Perceived severity showed a borderline association (OR = 1.158, 95% CI: 0.983–1.363, *P* = 0.079). For the hypertensive group, independent positive factors included self-efficacy (OR = 1.464, 95% CI: 1.209–1.775, *P* < 0.001), marital status (widowed/divorced vs. unmarried: OR = 4.960, 95% CI: 1.101–22.348, *P* = 0.037), perceived susceptibility (OR = 1.163, 95% CI: 1.016–1.332, *P* = 0.029), and lifestyle score (OR = 1.089, 95% CI: 1.019–1.164, *P* = 0.012). Age group 18–39 years was negatively associated with intervention intention (OR = 0.103, 95% CI: 0.019–0.560, *P* = 0.009). Occupation in government/institution showed a borderline negative association (OR = 0.202, 95% CI: 0.039–1.046, *P* = 0.057).

**Table 3:** Multivariate logistic regression analysis of factors associated with health intervention intention in the total population (*N* = 1,050)

Factor	β	SE	OR	95% CI	*P*-value
**Family history of hypertension (No vs. Yes)**	1.113	0.29	3.043	1.725–5.369	< 0.001
**Lifestyle questionnaire score**	0.239	0.021	1.269	1.218–1.323	< 0.001
**HBM dimensions**					
Perceived susceptibility	0.071	0.037	1.074	0.998–1.155	0.056
**Perceived severity**	0.075	0.031	1.078	1.014–1.146	0.017
Perceived benefits	0.05	0.043	1.051	0.966–1.144	0.249
Perceived barriers	-0.034	0.034	0.966	0.905–1.033	0.312
**Health motivation**	0.12	0.047	1.128	1.029–1.236	0.01
Self-efficacy	0.087	0.053	1.091	0.984–1.211	0.099

Binary logistic regression analysis with health intervention intention (willing/very willing vs. unwilling) as the dependent variable. Independent variables were entered in three blocks: Block 1 (demographic and clinical variables), Block 2 (HBM dimensions), and Block 3 (lifestyle questionnaire score). All categorical variables use the last category as the reference group. For family history of hypertension: 1 = Yes, 2 = No (reference). Health status reference category is "Poor" (coded as 4). Statistically significant ORs (*P* < 0.05) are shown in bold

*OR* Odds Ratio, *CI* Confidence Interval, *HBM* Health Belief Model

## Discussion

Significant multidimensional disparities exist in demographic characteristics, health cognition, health beliefs, and intervention intention between prehypertensive and hypertensive populations in Chengdu, consistent with the epidemiological features of hypertension in Chinese adults [4, 33]. This large-sample study validated the applicability of the Health Belief Model (HBM) in explaining health behavior intention among hypertensive and prehypertensive individuals in the Chinese community context [34, 35]. Lifestyle score was the strongest universal positive correlate of intervention intention across all populations, and it was similarly associated with intervention intention in both groups.This finding aligns with the International Society of Hypertension (ISH) position paper, which strongly recommends lifestyle modifications as the “first-line strategy to prevent and control hypertension in adulthood” [36].

In contrast, self-efficacy and perceived severity were more strongly associated with intervention intention in hypertensive patients, while the associations were weaker in prehypertensive individuals [34, 35]. This pattern is consistent with Wang et al., who found that HBM-based health education significantly improved self-efficacy (MD = 12.4, *P* < 0.001) and perceived severity (MD = 3.1, *P* < 0.001) in hypertensive patients compared to routine care [35]. Additionally, a systematic review by Islam et al. confirmed that across 24 studies (*n* = 6,106), “perceived susceptibility, severity, and self-efficacy are positively associated with BP reduction, whereas perceived barriers have a negative impact on adherence” [37].Stratified analysis revealed that the prehypertensive group can be characterized as a “lifestyle-sensitive” population, whereas the hypertensive group can be characterized as a “disease perception-sensitive” population. These findings are consistent with the view that health intervention intentions are associated with a combination of demographic, clinical, lifestyle, and psychological factors, with clear stage-specific patterns, providing evidence for targeted hypertension prevention strategies [35, 38].

The lifestyle score of hypertensive patients was significantly higher than that of prehypertensive individuals, which is consistent with a “treatment adherence effect”—a clinical diagnosis may be associated with more active lifestyle adjustments under medical guidance. The strong positive correlation between lifestyle and intervention intention in the prehypertensive group suggests that lifestyle improvement is closely associated with greater proactive health management willingness. From a clinical perspective, lifestyle intervention should be considered central to prehypertension control, with targeted plans tailored to demographic characteristics (e.g., higher education, government/institution occupations, higher income). Even for hypertensive patients with relatively healthy lifestyles, continuous lifestyle guidance remains necessary [39–41].

The differentiated roles of health motivation and self-efficacy across the two groups represent another key finding of this study, reflecting stage-specific differences in health psychological factors during hypertension development [40]. In hypertensive patients, these factors were core independent predictors of intervention intention, consistent with the idea that long-term medication adherence and sustained lifestyle adjustments are related to internal motivation and self-efficacy. Their clinical experience may deepen understanding of hypertension severity, making health motivation more targeted and persistent. This finding is supported by Yang et al., who validated the six-factor structure of the Hypertension Belief Assessment Tool (HBAT) in 325 Chinese hypertensive patients, demonstrating good model fit (CFI = 0.952, RMSEA = 0.068) and high internal consistency (Cronbach’s α = 0.803), confirming the robustness of HBM dimensions—including health motivation and self-efficacy—in measuring hypertension-related beliefs in the Chinese population [42]. In contrast, prehypertensive individuals have not experienced the clinical course, leaving their disease risk perception vague and internal drive insufficient. This finding aligns with Kashani et al., who studied 200 prehypertensive individuals and found that self-efficacy (β = -0.25, *P* < 0.001) and perceived severity (β = 0.32, *P* < 0.001) were the strongest predictors of blood pressure control behaviors, while perceived benefits and barriers did not significantly impact behaviors [38]. These observations suggest that psychological interventions may be usefully integrated into routine hypertension management. Cognitive behavioral therapy, peer support groups, and personalized health education may enhance patients‘ internal motivation and confidence, potentially improving intervention persistence and blood pressure control [39, 40].

The heterogeneous influencing patterns also call for stratified community hypertension management [33]. The prehypertensive group’s “lifestyle sensitivity” and high model explanatory power (Nagelkerke R² = 0.849) indicate relatively concentrated influencing factors, enabling targeted interventions centered on lifestyle improvement while reducing practical and psychological barriers. Given their higher education and better economic conditions, community health services could adopt digital tools, personalized counseling, and community lectures to convey progression risks and early intervention benefits. Convenient services—free blood pressure measurement, personalized diet and exercise plans—may reduce practical barriers and strengthen intervention willingness.

For the hypertensive group’s “disease perception sensitivity,” the moderate model explanatory power (Nagelkerke R²=0.544) suggests multifactorial influences, requiring comprehensive, multidimensional strategies. Beyond lifestyle guidance, enhancing health motivation and self-efficacy may be crucial, alongside attending to social support and health literacy [33, 35]. Community health services could establish integrated management systems combining physical and psychological intervention, regularly assess health beliefs, and provide targeted education. Building social support networks—family and peer support—may alleviate treatment fatigue and improve adherence [4, 35, 38].

These findings have important implications for China’s chronic disease prevention system. Hypertension remains the most common chronic disease, with high prevalence and low awareness, treatment, and control rates [4]. Prehypertension—a key window for early cardiovascular prevention—affects a large population, with 65–70% progressing to clinical hypertension within 10–15 years [4, 39], yet remains underemphasized. This study confirms that prehypertensive individuals have significant deficits in health cognition and intervention willingness, which may explain low early intervention rates. Therefore, incorporating prehypertension into community chronic disease management with specialized screening and intervention systems is worth considering.

The heterogeneous characteristics of both populations support moving away from traditional unified hypertension management models toward stratified systems with distinct indicators, priorities, and approaches [33]. For prehypertensive individuals, evaluation should focus on lifestyle improvement and regular blood pressure monitoring, with priorities on lifestyle guidance and health education—salt control, physical activity, dietary adjustment [37, 43, 44]. Reducing daily salt intake by 1 g in China could prevent nearly 9 million cardiovascular events by 2030 [45]. For hypertensive patients, evaluation should cover blood pressure control, medication adherence, health beliefs, and lifestyle, with comprehensive management integrating drug treatment, lifestyle guidance, and psychological intervention [4, 33, 35]. This stratified approach may improve efficiency, conserve resources, and enhance community chronic disease management.

This study also advances HBM application in Chinese chronic disease research. Although widely applied, most studies lack exploration of heterogeneous model application across subgroups [37, 41]. This study confirms that HBM effectively predicts intervention intention in both populations, with dimensions exhibiting group-specific predictive effects. The cross-cultural applicability of HBM is supported by international studies, such as its use in Ghana [46]. Notably, perceived severity and perceived benefits showed no significant correlation with intervention intention in our study, possibly reflecting the “double-edged sword” effect of perceived severity—excessive risk perception may lead to anxiety, while insufficient perception may lead to neglect—or the population’s relatively simplistic understanding of intervention benefits. Researchers should consider China’s cultural background and refine model dimensions accordingly [40]. For instance, perceived severity could be distinguished into moderate risk perception (potentially conducive) and excessive anxiety (potentially detrimental), while perceived benefits could include short-term (e.g., improved physical comfort) and long-term (e.g., reduced cardiovascular risk) benefits [35–37]. Emerging digital health interventions, such as mHealth programs for young adults with prehypertension, offer promising avenues for operationalizing HBM constructs through real-time behavioral support [47].

The findings should be interpreted within Sichuan Province’s hypertension prevention context. According to the 2024 Report on Population Health Status and Key Diseases in Sichuan Province, based on 2023 chronic disease surveillance, hypertension prevalence among residents aged ≥ 18 years in Sichuan Province reached 33.55%, higher than the national average [28]. A retrospective study in southern Sichuan further reported a hypertension prevalence of 38.5% and demonstrated that regular physical activity significantly reduced hypertension risk (OR = 0.682) [48]. Health cognition and intervention willingness in Chengdu are directly relevant to provincial hypertension management effectiveness. These findings support targeted stratified intervention strategies to reduce clinical hypertension incidence, improve control rates, and alleviate cardiovascular burden. Regionally tailored policies should account for local characteristics—high-salt and high-spice dietary habits, lifestyle patterns, and population structure. Early intervention in prehypertensive populations and comprehensive management of hypertensive patients are essential. Systematic reviews further emphasize integrating evidence-based guidelines into local prevention frameworks, particularly for high-risk populations [49].

This study has several limitations. First, the cross-sectional design precludes causal inference; prospective cohort studies are needed. Second, convenience sampling from community health centers and hypertension clinics may have oversampled health-conscious individuals, potentially inflating health awareness and intervention willingness relative to the general population. This limits generalizability of absolute estimates, but comparative findings between groups are less affected as any bias would apply similarly to both groups. Future multi-center studies with stratified random sampling are warranted.Third, self-reported questionnaires may introduce recall and social desirability bias; objective measurement tools (e.g., accelerometers, ambulatory blood pressure monitoring) would improve accuracy.Fourth, the present study did not explore mediating or moderating effects among variables (including HBM dimension interactions and mediating pathways), and common method variance due to the cross-sectional design may inflate observed associations; future studies using structural equation modeling are recommended to elucidate these mechanisms and confirm discriminant validity and causal pathways. In addition, the wide confidence interval for family history in the prehypertensive subgroup suggests statistical instability; this estimate should be interpreted cautiously. Fifth, important factors such as social support and health literacy were not included in the current assessment system, despite their well-documented roles in chronic disease management; incorporating these variables would allow for more comprehensive analysis.

## Conclusions

Prehypertensive and hypertensive populations in China exhibit marked heterogeneity in factors influencing health intervention intention, underscoring the need for stage-specific, targeted strategies in community practice. For the prehypertensive group, interventions should prioritize lifestyle improvement and reducing practical and psychological barriers through diversified health education and convenient services. For the hypertensive group, beyond standardized drug treatment and lifestyle guidance, integrating psychological intervention to enhance health motivation and self-efficacy is essential, alongside building comprehensive medical, psychological, and social support systems. Implementation of these targeted strategies may enhance proactive health management willingness, promote standardized hypertension control, and reduce cardiovascular burden. Furthermore, China’s chronic disease prevention system could be strengthened by establishing, precise hypertension management models and including prehypertension within community chronic disease management, enabling whole-process care from prevention to treatment. This study provides an evidence-based foundation for these strategies, with important implications for improving chronic disease management in China and advancing the Healthy China initiative.

## Supplementary Information

Supplementary Material 1: Table 1. Multivariate logistic regression analysis of factors associated with health intervention intention in prehypertensive and hypertensive groups. Only variables with *P* < 0.10 are shown for brevity. All categorical variables use the last category as the reference group. HBM dimensions represent perceived susceptibility, perceived severity, perceived benefits, perceived barriers, health motivation, and self-efficacy, respectively. OR = Odds Ratio; CI = Confidence Interval. Table 2. Variable coding scheme for the multivariable logistic regression analysis. All categorical variables used in the regression models are listed with their numeric codes, value labels, and reference categories (indicated by *). Continuous variables (perceived susceptibility, perceived severity, perceived benefits, perceived barriers, health motivation, self-efficacy, and lifestyle score) were entered with their actual scores. Table 3. Full multivariate logistic regression results including non-significant demographic variables (Total population, *N* = 1,050). This table presents the complete regression results for all demographic and clinical variables. Only variables with *P* > 0.05 are listed here; the main Table 3. in the manuscript includes significant predictors (family history of hypertension, lifestyle score, perceived severity, health motivation) and theoretically important HBM dimensions. All categorical variables use the last category as the reference group. All monetary values are in Chinese Yuan (CNY). Table 4. Detailed independent-samples t-test results for HBM dimension scores between prehypertensive and hypertensive groups. Data are presented as mean ± standard deviation (SD). 95% CI = 95% confidence interval of the mean difference (hypertensive group minus prehypertensive group). P-values are two-tailed. This table supports the statistical significance reported in the Results section (all *P* < 0.05), despite some overlap of error bars in Fig. 2. Supplementary Note: (1) All figures are in TIFF format with a resolution of ≥ 300dpi (RGB color mode) and a single file size ≤ 20 MB; (2) All tables are in three-line table format, with continuous variables expressed as mean ± SD and categorical variables as n (%); (3) Statistical significance: *P < 0.05, **P < 0.01, all tests are two-tailed.

## Data Availability

The datasets generated and analyzed during the current study are available from the corresponding author on reasonable request.
