# Supplementary material for: Disease risk perception, health intervention intention and their influencing factors among prehypertensive and hypertensive populations: a cross-sectional study with 1050 participants
Source: BMC Public Health. 2026 Jun 25;26:2553. doi: 10.1186/s12889-026-28231-1 (PMC13555877; doi:10.1186/s12889-026-28231-1)
Supplement: Supplementary file 1 [file 12889_2026_28231_MOESM1_ESM.zip › Supplementary Materials/Supplementary Table.docx]

**Supplementary Table 1** Multivariate logistic regression analysis of factors associated with health intervention intention in prehypertensive and hypertensive groups

| **Group** | **Variable** | **β** | **SE** | **OR** | **95% CI** | **P-value** |
| --- | --- | --- | --- | --- | --- | --- |
| **Prehypertension (n = 525)** | Family history of hypertension (Yes vs. No) | 3.116 | 0.597 | 22.557 | 7.007–72.622 | <0.001 |
|  | Health motivation | 0.335 | 0.100 | 1.398 | 1.150–1.700 | 0.001 |
|  | Lifestyle questionnaire score | 0.427 | 0.050 | 1.533 | 1.388–1.692 | <0.001 |
|  | Age group (40–59 vs. ≥60) | 1.904 | 0.898 | 6.710 | 1.154–39.014 | 0.034 |
|  | Occupation (Government/institution vs. Retired) | 1.369 | 0.661 | 3.932 | 1.076–14.364 | 0.038 |
|  | Health status ("General"vs. "Poor") | 2.578 | 1.514 | 13.169 | 0.678–255.914 | 0.089 |
|  | Perceived severity (C2) | 0.146 | 0.083 | 1.158 | 0.983–1.363 | 0.079 |
| **Hypertension**  **(n = 525)** | Self-efficacy | 0.382 | 0.098 | 1.464 | 1.209–1.775 | <0.001 |
|  | Marital status (Widowed/divorced vs. Unmarried) | 1.601 | 0.768 | 4.960 | 1.101–22.348 | 0.037 |
|  | Perceived susceptibility | 0.151 | 0.069 | 1.163 | 1.016–1.332 | 0.029 |
|  | Lifestyle questionnaire score | 0.085 | 0.034 | 1.089 | 1.019–1.164 | 0.012 |
|  | Age group (18–39 vs. ≥60) | -2.271 | 0.863 | 0.103 | 0.019–0.560 | 0.009 |
|  | Occupation (Government/institution vs. Retired) | -1.599 | 0.839 | 0.202 | 0.039–1.046 | 0.057 |

**Note: OR = Odds Ratio; CI = Confidence Interval. Only variables with P < 0.10 are shown for brevity. All categorical variables use the last category as the reference group.*

### Supplementary Table 2 Variable coding scheme for the multivariable logistic regression analysis

| **Variable** | **Code**  **in analysis** | **Value label** | **Reference** |
| --- | --- | --- | --- |
| Health intervention intention (Y1) | 0 | Unwilling | * |
|  | 1 | Willing |  |
| Gender (X1) | 1 | Male |  |
|  | 2 | Female | * |
| Age group (X2) | 1 | 18～<40 years |  |
|  | 2 | 40～<60 years |  |
|  | 3 | ≥60 years | * |
| BMI (X3) | 1 | <24 kg/m² |  |
|  | 2 | 24～<28 kg/m² |  |
|  | 3 | ≥28 kg/m² | * |
| Education level (X4) | 1 | Primary school or below |  |
|  | 2 | High school/technical |  |
|  | 3 | College or above | * |
| Occupation (X5) | 1 | Government/institution |  |
|  | 2 | Enterprise/business |  |
|  | 3 | Retired | * |
| Family monthly income  (CNY)(X6) | 1 | <2500 |  |
|  | 2 | 2500～<4999 |  |
|  | 3 | 5000～<7500 |  |
|  | 4 | ≥7500 | * |
| Marital status (X7) | 1 | Unmarried |  |
|  | 2 | Married/cohabiting |  |
|  | 3 | Widowed/divorced | * |
| Living status (X8) | 1 | Living alone |  |
|  | 2 | With family (non-parents/children) |  |
|  | 3 | With children/parents | * |
| Comorbid chronic diseases(X9) | 1 | None |  |
|  | 2 | Diabetes |  |
|  | 3 | Other diseases | * |
| Self-rated health status (X10) | 1 | Very good |  |
|  | 2 | Good |  |
|  | 3 | General |  |
|  | 4 | Poor | * |
| Family history of hypertension (X11) | 1 | Yes |  |
|  | 2 | No | * |
| Perceived susceptibility (X12) | Continuous | Actual score | N/A |
| Perceived severity (X13) | Continuous | Actual score | N/A |
| Perceived benefits (X14) | Continuous | Actual score | N/A |
| Perceived barriers (X15) | Continuous | Actual score | N/A |
| Health motivation (X16) | Continuous | Actual score | N/A |
| Self-efficacy (X17) | Continuous | Actual score | N/A |
| Lifestyle score (X18) | Continuous | Actual score | N/A |

Note: * indicates the reference category. BMI = body mass index.

**Supplementary Table 3** Full multivariate logistic regression results including non‑significant demographic variables (Total population, N = 1,050)

| **Factor** | **β** | **SE** | **OR** | **95% CI** | **P-value** |
| --- | --- | --- | --- | --- | --- |
| **Demographic and clinical variables** |  |  |  |  |  |
| Gender (Female vs. Male) | -0.159 | 0.323 | 0.853 | 0.453–1.606 | 0.623 |
| Age group (≥60 vs. 18～<40) | 0.265 | 0.492 | 1.303 | 0.497–3.414 | 0.59 |
| Age group (≥60 vs. 40～<60) | -0.231 | 0.424 | 0.794 | 0.346–1.822 | 0.587 |
| Education (College+ vs. Junior high) | 0.125 | 0.356 | 1.133 | 0.563–2.279 | 0.727 |
| Education (College+ vs. High school) | -0.659 | 0.357 | 0.517 | 0.257–1.041 | 0.065 |
| Marital status (Widowed/divorced vs. Unmarried) | 0.001 | 0.54 | 1.001 | 0.348–2.880 | 0.998 |
| Marital status (Widowed/divorced vs. Married/cohabiting) | -0.189 | 0.495 | 0.828 | 0.314–2.184 | 0.702 |
| Monthly income (≥7500 vs. <2500) | 0.036 | 0.655 | 1.037 | 0.287–3.746 | 0.957 |
| Monthly income (≥7500 vs. 2500～<5000) | -0.587 | 0.462 | 0.556 | 0.225–1.375 | 0.204 |
| Monthly income (≥7500 vs. 5000～<7500) | -0.173 | 0.325 | 0.841 | 0.445–1.590 | 0.595 |
| Occupation (Retired vs. Government/institution) | -0.001 | 0.378 | 0.999 | 0.476–2.094 | 0.997 |
| Occupation (Retired vs. Enterprise/business) | -0.026 | 0.359 | 0.974 | 0.482–1.969 | 0.942 |
| Living status (With children/parents vs. Living alone) | -0.111 | 0.423 | 0.895 | 0.391–2.049 | 0.793 |
| Living status (With children/parents vs. With family, non‑parents/children) | 0.22 | 0.31 | 1.246 | 0.679–2.288 | 0.477 |
| Health status (Poor vs. Very good) | 0.919 | 0.789 | 2.507 | 0.534–11.765 | 0.244 |
| Health status (Poor vs. Good) | 0.08 | 0.608 | 1.083 | 0.329–3.571 | 0.895 |
| Health status (Poor vs. General) | -0.813 | 0.631 | 0.444 | 0.129–1.529 | 0.198 |
| Comorbidities (Other vs. None) | -0.827 | 0.685 | 0.437 | 0.114–1.675 | 0.227 |
| Comorbidities (Other vs. Diabetes) | -0.946 | 0.711 | 0.388 | 0.096–1.565 | 0.183 |

*Note: Only variables with P > 0.05 are listed here. The main Table 3 in the manuscript includes significant predictors (family history, lifestyle score) and theoretically important HBM dimensions. All categorical variables use the last category as the reference group.All monetary values are in Chinese Yuan (CNY).*

## **Supplementary Table 4.**  Detailed t-test results for HBM dimension scores between prehypertensive and hypertensive groups

| **HBM**  **Dimension** | **Group** | **Mean** | **SD** | **95% CI** | **t** | **P-value** |
| --- | --- | --- | --- | --- | --- | --- |
| **Perceived Susceptibility** | Prehypertension (n=525) | 15.21 | 4.220 | (-1.362,-0.308) | -3.108 | 0.002 |
|  | Hypertension (n=525) | 16.05 | 4.277 |  |  |  |
| **Perceived**  **Severity** | Prehypertension (n=525) | 13.78 | 4.081 | (-2.808, -1.508) | -6.514 | <0.001 |
|  | Hypertension (n=525) | 15.94 | 6.402 |  |  |  |
| **Perceived**  **Benefits** | Prehypertension (n=525) | 25.15 | 3.123 | (2.060, 3.057) | 10.071 | <0.001 |
|  | Hypertension (n=525) | 22.59 | 4.911 |  |  |  |
| **Perceived**  **Barriers** | Prehypertension (n=525) | 25.01 | 3.498 | (4.969, 6.288) | 16.737 | <0.001 |
|  | Hypertension (n=525) | 19.38 | 6.866 |  |  |  |
| **Health**  **Motivation** | Prehypertension (n=525) | 27.88 | 3.381 | (0.194, 1.174) | 2.740 | 0.006 |
|  | Hypertension (n=525) | 27.19 | 4.612 |  |  |  |
| **Self-efficacy** | Prehypertension (n=525) | 18.19 | 3.014 | (-0.758, -0.004) | -1.983 | 0.048 |
|  | Hypertension (n=525) | 18.57 | 3.209 |  |  |  |

*Note:*Abbreviations: SD, standard deviation; CI, confidence interval. Mean difference = hypertensive – prehypertensive. P values are two-tailed. The table supports the statistical significance reported in the Results section (all P < 0.05), despite some overlap of error bars in Figure 2.
